# Fatty Acid Synthase Impacts the Pathobiology of *Candida parapsilosis In Vitro* and during Mammalian Infection

**DOI:** 10.1371/journal.pone.0008421

**Published:** 2009-12-22

**Authors:** Long Nam Nguyen, David Trofa, Joshua D. Nosanchuk

**Affiliations:** 1 Department of Medicine (Division of Infectious Diseases), Albert Einstein College of Medicine, New York, New York, United States of America; 2 Department of Microbiology and Immunology, Albert Einstein College of Medicine, New York, New York, United States of America; University of Texas-Houston Medical School, United States of America

## Abstract

Cytosolic fungal fatty acid synthase is composed of two subunits α and β, which are encoded by Fas1 and Fas2 genes. In this study, the Fas2 genes of the human pathogen *Candida parapsilosis* were deleted using a modified SAT1 flipper technique. *CpFas2* was essential in media lacking exogenous fatty acids and the growth of Fas2 disruptants (Fas2 KO) was regulated by the supplementation of different long chain fatty acids, such as myristic acid (14∶0), palmitic acid (16∶0), and Tween 80, in a dose-specific manner. Lipidomic analysis revealed that Fas2 KO cells were severely restricted in production of unsaturated fatty acids. The Fas2 KO strains were unable to form normal biofilms and were more efficiently killed by murine-like macrophages, J774.16, than the wild type, heterozygous and reconstituted strains. Furthermore, Fas2 KO yeast were significantly less virulent in a systemic murine infection model. The Fas2 KO cells were also hypersensitive to human serum, and inhibition of *CpFas2* in WT *C. parapsilosis* by cerulenin significantly decreased fungal growth in human serum. This study demonstrates that *CpFas2* is essential for *C. parapsilosis* growth in the absence of exogenous fatty acids, is involved in unsaturated fatty acid production, influences fungal virulence, and represents a promising antifungal drug target.

## Introduction

*Candida parapsilosis* is a major human pathogen that has dramatically increased in significance and prevalence over the past two decades [1], [2]. At present, *C. parapsilosis* is among the most common *Candida* species causing invasive disease worldwide and it is the most common non-*albicans* species in some countries. *C. parapsilosis* is a frequent commensal of epithelial and mucosal tissues and the fungus is often isolated from hospital environments and from the hands of healthcare workers [3]. The pathogenesis of the fungus is attributed to a number of virulence factors, including its ability to adhere to host cells, form biofilm, and secrete hydrolytic enzymes such as lipases, aspartic proteinases and phospholipases [2]. Unfortunately, despite its increasing importance and prevalence, little is known about the molecular mechanisms of *C. parapsilosis* virulence. Recently, we demonstrated that a secreted lipase was a major virulence determinant in *C. parapsilosis* [4]. However, the relationship between lipid metabolism and fungal virulence is poorly understood.

The *de novo* formation of fatty acids is essential for diverse organisms, and is typically performed by a complex of enzymes. Three major fatty acid synthesis systems have been elucidated. The majority of eukaryotes and certain advanced prokaryotes, such as *Mycobacterium* spp., *Nocardia* spp. and *Corynebacterium* spp., use the type I fatty acid synthesis (FASI) system, which utilizes larger multifunctional enzymes, while most bacteria use the FASII system, composed of smaller separate enzymes [5]. A third fatty acid synthesis system has been identified in certain parasites, including *Trypanosoma brucei*, *Leishmania major* and *Trypanosoma cruzi*, that involves membrane-bound elongases for the synthesis of aliphatic chains [6]. Similar to bacterial FASII, the enzymes involved in fatty acid synthesis in these parasites are smaller in structure. Although the enzymes involved in the different FAS systems are conserved between kingdoms, the organization of genes encoding the enzymes varies. In bacteria and parasites genes coding for fatty acid synthesis enzymes are in separate open reading frames with each encoding an individual catalytic enzyme [5]. The genes coding fungal FAS enzymes are generally composed of two open reading frames, each encoding an enzyme with at least three catalytic domains. Fungal fatty acid synthesis enzymes form a 2.6-MD heterododecameric complex including six subunits α and six subunits β, which are encoded by the genes Fas1 and Fas2, respectively, whereas mammalian FAS consists of a 270-kD polypeptide chain (including seven domains) that assemble into homodimers [7], [8].

Despite the variation in structural organization, the individual reaction steps of fatty acid biosynthesis are conserved. The vital roles of the fatty acid synthases make it a promising target for antifungal and antibacterial treatment. Importantly, bacterial and fungal FAS(s) are distinct from human ones, which makes this pathway an attractive target. For example, intensive research has been focused on targeting fatty acid synthesis of mycobacteria [9], [10], [11]. However, recent data demonstrates that certain bacteria, such as streptococci and staphylococci, can obtain sufficient exogenous fatty acids to overcome FASII inhibition *in vivo* [12]. In fungi, Fas2 inhibition can significantly reduce the pathogenesis of *Cryptococcus neoformans* [13] and *Candida albicans* [14], [15]. In fact, deletion of the Fas2 genes in *C. albicans* impaired the pathogenesis of this fungus in both a rat oropharyngeal and a systemic mouse infection model [14], [15]. These studies suggest that Fas2 is a possible broad spectrum antifungal drug target.

In this report, we characterize the *C. parapsilosis* Fas2 genes and generate heterozygous, homozygous and reconstituted Fas2 mutants. We found that *CpFas2* was required for fungal growth in standard medium without exogenous fatty acids, and that the expression of Fas2 genes was repressed by the presence of fatty acids, but up-regulated in the presence of glucose as the sole carbon source. We additionally analyzed the lipid profile of wild type and disrupted yeast cells and found that unsaturated fatty acids were significantly reduced in Fas2 KO yeast. *CpFas2* was also required for normal biofilm formation. Moreover, deletion of *CpFas2* resulted in attenuated virulence in a systemic murine infection model.

## Material and Methods

### Strains and Culture Conditions

*C. parapsilosis* strains were maintained at −80°C in 35% glycerol. If not otherwise mentioned, the strains were grown in either YPD (1% yeast extract, 2% bactopeptone, 2% glucose), or YPDT40 (YPD plus 1% Tween 40, 0.01% myristic and stearic acids). Strains generated and used in this study are listed in table 1.

**Table 1:** *C. parapsilosis* strains generated and used in this study.

Name	Genotype	Reference
GAL1A	Wild type (WT)	[4]
Fas2 HET^R^ (Nou^R^)	*CpFas2/ΔCpFas2::SAT1-FLIP*	This study
Fas2 HET (Nou^S^)	*CpFas2/ΔCpFas2::FRT*	This study
Fas2 KO^R^ (Nou^R^)	*ΔCpfas2/Δfas2::SAT1-FLIP*	This study
Fas2 KO (Nou^S^)	*ΔCpfas2/Δfas2::FRT*	This study
Fas2 RE (Nou^S^)	*CpFas2/ΔCpfas2::FRT*	This study

R Resistant to nourseothricin (Nou^R^); ^S^Sensitive to nourseothricin (Nou^S^).

### Generation of Disruption Construct pSFS2Fas2

The pSFS2Fas2 plasmid was used to disrupt the entire open reading frames of the Fas2 genes of 5655 nucleotides. A 481 bp upstream fragment of the Fas2 gene was cloned by PCR from gDNA with the use of the primer pair (C.pFAS2upF: CGGGGTACCCTAGTGTCGTGTCGCATCC (*Kpn*I); C.pFAS2upR: CCGCTCGAGCGCGTAGGTAAGTGATGATG (*Xho*I)). The PCR fragment was ligated into pGEMT, then transferred to pSFS2 [16] and opened with *Kpn*I and *Xho*I to yield pSFS2upFas2 plasmid. A 423 bp fragment downstream of the Fas2 gene was similarly cloned by PCR from gDNA with the use of the primer pair (C.pFAS2doF: TCCCCGCGGAAAAATCAACAAAGCCTCAAG (*Sac*II); C.pFAS2doR: CCCGAGCTCCTATGATTGGTGTTTGCGG (*Sac*I)) and then subcloned into pSFS2upFas2, which was digested with *Sac*I and *Sac*II to generate the pSFS2Fas2 disruption plasmid.

### *Candida parapsilosis* Transformation and Generation of CpFas2 Deletion Mutants

*C. parapsilosis* cells were transformed by electroporation as previously described [4] with minor modifications. Briefly, a single colony was inoculated in 50 ml YPD medium overnight with shaking at 150 rpm and 30°C. The cells were collected and centrifuged at 1000 g for 5 min in a 50 ml falcon tube. The cells were suspended in 45 ml TE buffer (10 mM Tris-Cl, 1 mM EDTA, pH 7.5) containing 100 mM lithium acetate and incubated for 45 min at 30°C with gentle shaking. After the addition of 0.45 ml of 1 M DTT (Dithiothreitol) and an additional 15 min of shaking, the cells were washed three times with ice-cold water and once with 1 M sorbitol. Finally, the cells were diluted in 150 µl of 1 M sorbitol and kept on ice. For a single transformation, 40 µl of the cell suspension was used with 10 µl of DNA (10 µg).

To generate the heterozygous strain (Fas2 HET), the clinical isolate *C. parapsilosis* strain GA1 (WT) was transformed with 10 µg of ethanol-precipitated DNA plasmid of pSFS2Fas2, which was digested overnight with *Kpn*I and *Sac*I. Transformants were analyzed by Southern blot. To eliminate the nourseothricin selection marker, the mutant strain was induced with 1% maltose in YNB (yeast nitrogen base, without glucose) medium overnight. Nourseothricin sensitive colonies were selected with low concentration of selection marker (20–25 µg/ml) in YPD medium as previously described [4]. This strain was subsequently used to generate the homozygous disruptants (Fas2 KO). The transformation procedures as described for the Fas2 HET strain were repeated with the pSFS2Fas2 plasmid. Transformants were selected and analyzed by Southern blot. The induction in maltose YNB medium was performed as described above to eliminate the nourseothricin selection marker. Cells were then cultivated in YPD with or without fatty acids.

### Generation of Construct for Reconstituted Fas2 Gene

As described in the results below, the Fas2 KO strain is autotrophic for fatty acids. Hence, the Fas2 gene could be employed as a selection marker for the mutant strain. We cloned the entire Fas2 gene and transformed it into the Fas2 KO strain to generate the reconstituted strain (Fas2 RE), which harbors one copy of the Fas2 gene. A 6347 bp fragment including the entire Fas2 gene was cloned by PCR with the use of primer pair: Fas2ReF: CGGGGTACCTTCTTTGGAATCTAGTGTCGTGT; Fas2ReR CCGCTCGAG TGACTCTCACAGCTTGTTTTGTC. Since this is a long PCR product, the long expand Taq polymerase Kit (Roche) was used to avoid a mismatch. The PCR product then underwent ethanol precipitation and was transformed into the Fas2 KO strain. Transformants were regenerated and analyzed by Southern blot for the presence of Fas2 gene in the native locus. Transformants were grown in YPD without fatty acids.

### Southern Blot Analysis

Genomic DNA was isolated and digested with appropriate enzymes. DNA was then separated on 0.8% agarose gels and transferred to passively charged nilon membranes (Amersham). The membranes were hybridized with digoxigenin-11-dUTP labeled DNA probe that was amplified by PCR with the use of CpFAS2upF/R primers. Detection and visualization of DNA was performed per the manufacturer's instructions (DIG DNA labeling and detection Kit, Roche).

### Quantitative Real-Time PCR (qRT-PCR)

The WT *C. parapsilosis* strain was cultured overnight in YP (1% yeast extract, 2% peptone), YP plus fatty acids, and YP plus 2% glucose. Individual fatty acids tested were 0.1% (w/v) myristic acid (14∶0), stearic acid (18∶0), and oleic acid (18∶1). Additionally, Tween 40 and Tween 80 were used at 0.5% (v/v). Total RNA from 1 ml of the overnight cultures (approximately 10^8^ cells/ml) were isolated by using RNeasy Kit (Qiagen) per the manufacturer's protocol for yeast. One stranded cDNA was then synthesized using SuperScript III Kit (Invitrogen). To evaluate the quality and concentration of cDNA for qRT-PCR, serial 10 fold dilutions of each sample was prepared and tested with the use of the alpha-tubulin primer pair qTubAF (CAGAGCTGTTTGTATGTTGTCCA) and qTubAR (ATTCACCTTCTTCCATACCTTCAC). qRT-PCR was performed with the use of the following primer pairs: qFAS1F (AAGAACAAGGTATGGGTATGGACT) and qFAS1R (TTAGCACCACCAAAATGAACTG) for the Fas1 gene and the primer pair qFAS2F (AAGAACCGAGGAAATTTACAGAGA) and qFAS2R (GTACCGTGGAATGAAGCAACTC) for the Fas2 gene. A reaction of 10 µl of the cDNA, primers and the Sybr Green Supermix (Bio-rad) was run with 7900HT Fast Real-time PCR system and SDS2.3 software (AppliedBiosystems). Relative expression levels of Fas1 and Fas2 genes were evaluated by the comparative Ct value method. Expression levels of Fas1 and Fas2 genes of the WT *C. parapsilosis* grown in glucose and fatty acids containing medium were normalized to WT grown in YP medium (1% yeast extract, 2% peptone).

### Growth Assays

The growth rates of the WT *C. parapsilosis* and the constructed mutants were analyzed in liquid YPD, YPDT40, YNB (yeast nitrogen base) plus 50 mM glucose, and YNBT40 (YNB plus 50 mM glucose, 1% Tween 40, and 0.01% myristic and stearic acids), with all medium at pH ∼7.5. A single colony from the WT, HET, and RE strains was inoculated in 2.5 ml YPD medium, the KO strain was inoculated in 2.5 ml YPDT40 medium. These cultures were incubated overnight in an orbital shaker set at 150 rpm and 30°C. The yeast cells were washed three times with sterile Phosphate Buffer Saline (PBS) and counted using a hemacytometer. The experimental media were then inoculated with 5×10^6^ cells/ml in 24 well plates. The cell density (OD_600_) was read by a microtiter reader (Labsystem Multiskan MS) at the indicated times. Growth of Fas2 KO strains was also analyzed in fatty acid-containing medium that included YPD plus 0.001 to 0.1% (w/v) of myristic acid (14∶0), palmitic acid (16∶0), stearic acid (18∶0), oleic acid (18∶1) or 0.001 to 0.1% (v/v) of Tween 80. The growth of Fas2 KO strain in YPD was used as a negative control. The growth rates at different times were measured by OD_600_. The assays were performed in triplicate and repeated twice.

The growth of the WT and Fas2 KO strains in human serum was also analyzed. The WT and KO yeast cells were cultured overnight in YPD and YPDT40, respectively. Yeast in log phase growth were washed, diluted to 5×10^6^ cells/ml, inoculated in various concentrations of human serum (normal type, heat-inactivated) in culture tubes and incubated at 30°C with rotary shaking at 150 rpm. Aliquots were obtained at different times of growth and plated on YPD agar for the WT and YPDT40 for the KO to determine CFUs.

The impact of pH on the growth of the WT and Fas2 KO strains on YPD was also tested. Overnight cultures in YGP and YPDT40 broth were diluted with PBS to OD_600_ = 0.1, transferred to 24-well microtiter plates, and serially diluted 1∶10. Aliquots (2.5 µl) of diluted cultures were spotted onto YPDT40 solid medium with indicated pH values. Plates were incubated at 30°C and 37°C for 4 days.

### *C. parapsilosis* Cerulenin Test

Cerulenin is a specific inhibitor of fungal fatty acid synthase [17], [18]. Fungal susceptibility testing of *C. parapsilosis* WT and mutants to cerulenin was done by plate tests and a broth microdilution method. For the plate test, yeast cells were cultured overnight and washed with PBS. Two µls of 2×10^6^ cells of WT, HET, or RE were spotted on solid YPD plates containing 0, 0.5, 1, 1.5, and 2 µg/ml cerulenin. As cerulenin was diluted in DMSO, 1% DMSO was added to the plates. Plates were incubated for 2 days at 30°C. For the broth microdilution method, 2×10^6^ cells/ml were incubated in 1 ml RPMI 1640 medium containing various concentrations of cerulenin for 48 hours at 35°C. Determination of minimum inhibitory concentration (MIC90) was performed according to a standardized protocol from the Clinical Laboratory and Standards Institute (CLSI) [19].

### Biofilm Formation

For biofilm formation, 96- and 24-well polystyrene plates were obtained from Fisher Scientific and silicone elastomer sheets were obtained from Bentec Medical Corp [4]. The silicone surface was chosen due to its material similarity to biomedical indwelling devices. The silicone sheets were cut to fit inside the wells of 24- well plates. The 96-well plates and the 24-well plates with the silicone discs were blocked for non-specific interactions by overnight incubation with fetal calf serum (FCS) at 37°C. Overnight cultures of the C. *parapsilosis* strains were washed three times with PBS, and suspended at 10^7^ cells/ml in YNBT40 medium supplemented with 50 mM of glucose. The 96-well polysterene and 24-well silicone plates were incubated at 37°C without shaking for 48 hours with 100 µl or 1 ml of the yeast suspension, respectively. The plates were washed three times with 0.05% Tween 20 in Tris-buffered saline (TBS) to remove the non-adhered cells. Fungal cells that remained attached to the plastic surface were considered to have formed biofilm.

### Measurement of Biofilm Metabolic Activity by the XTT Reduction Assay

The metabolic activity assay was conducted using 2,3-bis(2-methoxy-4-nitro-5-sulfophenyl)-5-[(phenylamino) carbonyl]-2H-tetrazolium hydroxide (XTT) [20]. A mixed solution of 50 or 300 µl XTT (1 mg/ml in PBS) with 4 or 24 µl of menadione solution (1 mM in acetone; Sigma Aldrich) was added to each well of the 96- or 24- well plates, respectively. The plates were incubated for 5 hours at 37°C and colorimetric changes were read by a microtiter reader at 492 nm. Heat-killed cells and YNB medium without cells were used as negative controls.

### Microscopy of Biofilm

The mature biofilms were washed two times with TBS and stained with a solution of 1 mM FUN-1 and 25 µg/ml Concavalin A Alexa Flour 488 conjugates (Invitrogen) for 45 min at 37°C. Microscopic examinations of biofilms were performed using an Axiovert 200 M inverted microscope (Zeiss Axiovert 200 M inverted microscope). The objective used was 20X. To determine the structure of the biofilms, a series of horizontal (x-y) optical sections were taken throughout the biofilms with red and green fluorescence channels, which correspond to the emission of FUN1 and Alexa Fluor 488, respectively. Side views of the Z-stack images were performed with AxioVision 4.7 software.

### Phagocytosis and Killing Assays with Macrophage-Like Cells

The macrophage-like cell line J774.16 [21] was used to study the phagocytosis and phagocytic killing of the C. *parapsilosis* strains. Macrophages were cultured in DMEM with 10% heat-inactivated FCS and were plated at 10^5^ cells per well in 8-chamber polystyrene glass slides for the phagocytosis assays and both 24 well plates and 8-chamber polystyrene glass slides for the killing assay. Phagocytosis assays with *C. parapsilosis* were performed according to our described protocol [4]. Briefly, *C. parapsilosis* cells were grown overnight, washed 3 times in PBS, counted using a hematocytometer, stained with FITC (1 mg/ml in PBS) for 45 min, and suspended in DMEM medium. The cells were then co-incubated with the macrophage monolayer at an effector/target ratio of 15∶1. The co-cultures were incubated at 37°C for 1 hour. The wells were then washed three times with PBS to remove non-adherent *Candida* cells. Phagocytosis of each *C. parapsilosis* strain was assessed by counting the number of phagocytosed yeast cells in 200 macrophages. The phagocytic index was the ratio of the number of intracellular yeast cells to the number of macrophages counted.

Colony counts were made to determine the number of viable yeast cells after phagocytosis. *C. parapsilosis* cells co-incubated with macrophages for 2 hours were liberated from macrophages by forcibly disrupting the macrophages through pipetting in H_2_0 for 2 min. The yeast cells were collected, counted, and serial diluted prior to plating. Cells were plated in YPD (YPDT40 for the Fas2 KO strain) agar. Fungal killing was also assessed by microscopy. Co-cultures in 8 chambers-glass slides were washed twice with HBSS and stained with 0.01% acridine orange (Sigma-Aldrich) followed by staining with 0.05% crystal violet (Sigma-Aldrich) dissolved in 0.15 M NaCl. In each staining step, the slides were stained for 45 seconds and washed twice with HBSS. Finally, the slides were rinsed 3 times with PBS. Pictures were taken with an Axiovert 200 M inverted microscope. The objective used was 20X in red, green, and phase channels. Experiments were performed in triplicate and repeated twice.

### Fatty Acid Analysis

Total fatty acids from yeast cells grown at log phase were extracted as described by Schneiter and Daum [22] with modifications. Yeast cells from 2.5 ml overnight cultures were collected by centrifugation at 0.8 g, washed once with distilled water, suspended in 1 ml of cold methanol spiked with 10 µg of heptadecanoic acid (17∶0) (Sigma, St. Louis, MO, USA) as an internal standard, and disrupted by vortexing with 0.5 mm diameter glass beads. Fatty acids were extracted with chloroform/methanol (2∶1) for 1 hour at room temperature. Organic phase was collected in a glass tube and dried under stream of nitrogen. Fatty acid profiles were determined by a gas chromatograph as described by Stukey *et al*. [23].

### Lactate Dehydrogenase (LDH) Assay

Log phase yeast cells grown in YPD for WT and YPDT40 for Fas2 KO were collected and washed three times with PBS. A suspension of 10^6^ cells/ml in PBS was incubated with a final concentration of 0, 0.5 or 1 mM hydrogen peroxide at 37°C for 1 hour. The supernatants were collected by centrifugation and LDH activity measured by using a cytotoxity detection kit (Roche, Mannheim, Germany). LDH activity from supernatants of yeast cells incubated at different temperatures was similarly performed.

### Mouse Infection Models

A/J mice (female, 6–8 weeks of age; obtained from the National Cancer Institute) were inoculated intraperitoneally with 3×10^7^ WT or mutant yeast in 100 µl PBS. Animal care for this study was approved by the institutional Animal Care and Use Committee of Albert Einstein College of Medicine. CFU numbers were determined from the liver, kidneys, and spleen 3 and 5 days after infection by plating tissue homogenates on YPD agar.

### Statistical Analysis

The statistical analysis was performed using GraphPad Prism version 5.02 for Windows (GraphPad Software, San Diego California USA). The significance of differences between sets of data was determined by Newman-Keuls test or ANOVA according to the data.

## Results

### Generation of Fas2 Homozygous Knockout Strain by Using SAT1 Flipper System

**Figure 1:**
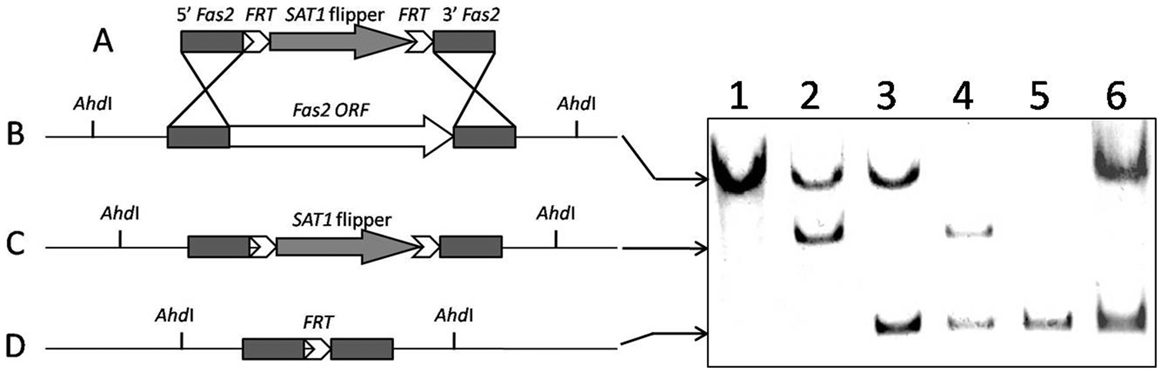
Disruption of Fas2 genes in *C. parapsilosis*. Schematic representation of disruption construct (A), genotype of wild type with Fas2 loci (B), disrupted locus with SAT1 cassette (C), and disrupted locus without SAT1 cassette (D). Southern blot analysis of wild type strain (lane1, genotype of B/B), heterozygous resistant strains (lane 2, genotype B/C), heterozygous non-resistant strain (lane 3, genotype B/D), homozygous resistant strain (lane 4, genotype C/D), homozygous non-resistant strain (lane 5, genotype D/D), and reconstituted strain (lane 6, genotype B/D). Southern blot probe was PCR amplified from the upstream fragment of plasmid pSFS2Fas2.

WT *C. parapsilosis* was transformed with the plasmid pSFS2Fas2 by electroporation resulting in more than 35 primary colonies. Eight colonies were randomly analyzed by Southern blot, which showed that one of the Fas2 alleles had been replaced by SAT1 flipper cassette (Figure 1, lane 2) in each transformant. In order to delete the remaining Fas2 gene and reuse the disruption construct, the SAT1 flipper cassette containing the selection marker was eliminated in the selected mutant to generate the sensitive heterozygous mutant (Figure 1, lane 3). This strain was used for the second transformation with the plasmid pSFS2Fas2 to create the homozygous mutant strains, which again harbored the SAT1 flipper cassette (Figure 1, lane 4). Initial screens failed to identify any transformed colonies. We subsequently plated potential homozygous disruptants onto YPDT40 and two independent transformations identified 3 mutants that were capable of growing in YPDT40 but not YGP. Southern blot analysis revealed that the native remaining Fas2 gene in the heterozygous mutant strains was completely disrupted, resulting in homozygous mutant strains (Figure 1, lane 4). The cassette was then eliminated to yield homozygous non-nourseothricin resistant, disrupted strains (Figure 1, lane 5). These complete disruptants were used to re-introduce the entire Fas2 open reading frame including approximately 400 base pairs fragments from upstream and downstream regions by transformation. Fifteen transformants were obtained by screening for the ability to grow on YPD medium and analyzed by Southern blot analysis to confirm the presence of one copy of Fas2 gene. Successful reconstituted mutants contained the Fas2 gene in the same locus as shown in the WT (Figure 1, lanes 1 and 6)

### *CpFas2* Is Essential for Fungal Growth in Fatty Acid-Free Medium, but Dispensable in Medium with Fatty Acids

**Figure 2:**
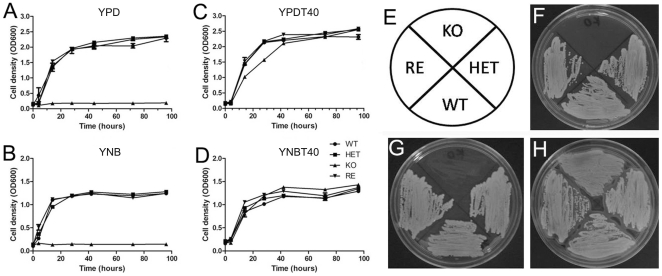
Growth rates of wild type (WT), heterozygous (HET), homozygous (KO), and reconstituted (RE) mutant strains. Yeast cell growth was compared in YPD (A), YPDT40 (YPD plus fatty acids) (B), YNB (C), and YNBT40 (YNB plus fatty acids) (D) broth. Growth of the *Candida* cells was measured by cell density at OD_600_. Illustration of the distribution of yeast strains on plates (E). Growth of WT, HET, KO, and RE in solid YPD without fatty acids (F), YPD with 0.5% (w/v) oleic acid (G), and YPD with 1% (v/v) Tween 40 and 0.01% (w/v) myristic and stearic aicds (H). Pictures were taken after 2 days of incubation at 30°C. Experiments were repeated twice with four replicates for each strain with the same results.

The growth rates of the fatty acid synthase heterozygous (HET), homozygous (KO) and reconstituted (RE) mutant strains were examined in YPD, YPD plus fatty acids (YPDT40), YNB, and YNB plus fatty acids (YNBT40). As shown in figure 2A and C, the KO strain was not able to propagate in YPD and YNB, although the cells were viable after >48 hours as determined by subsequent inoculation in YPDT40 (data not shown). The HET and RE strains, each of which contain a single copy of the Fas2 gene, exhibited growth rates similar to WT. All the mutant strains were able to grow in medium supplemented with fatty acids, although growth of the KO was slightly reduced initially. These data show that exogenous fatty acids can bypass the role of Fas2 in *C. parapsilosis* growth *in vitro*. The growth of these strains was also confirmed by inoculating yeast cells on YPD, YPD plus oleic acid and YPDT40 agar plates (Figure 2F–H).

### Growth of Fas2 KO Strains Is Dependent on Type and Amount of Different Fatty Acids

**Figure 3:**
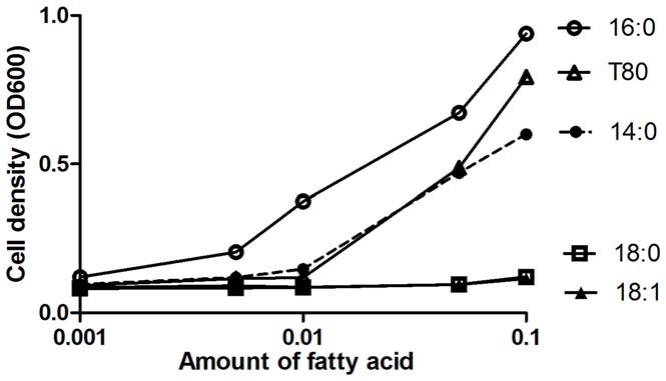
Growth dependence of Fas2 KO strain on different exogenous fatty acids in YPD. Different amounts of myristic (14∶0), palmitic (16∶0), stearic (18∶0), oleic (18∶1) acids, and T80 (Tween 80) were added to YPD broth. Yeast growth was determined by cell density after 48 hours at 30°C. The results were the mean of two independent experiments.

To gain additional insight into the requirement of fatty acids for KO growth, we tested the growth rates in YPD medium supplemented with different saturated and monounsaturated fatty acids at various concentrations. In the presence of myristic acid (14∶0), palmitic acid (16∶0), or Tween 80 as the fatty acid sources, the KO strains were able to grow in a concentration-dependent manner (Figure 3). However, KO yeast cells were unable to grow when stearic acid or oleic acid was added to the YPD medium. The results suggest that only certain fatty acids permit fungal growth in the absence of Fas2 function, and the growth rates of Fas2 KO strain is dependent upon fatty acid concentration.

### Transcription of Fatty Acid Synthase Genes Is Regulated by Carbon Sources

**Figure 4:**
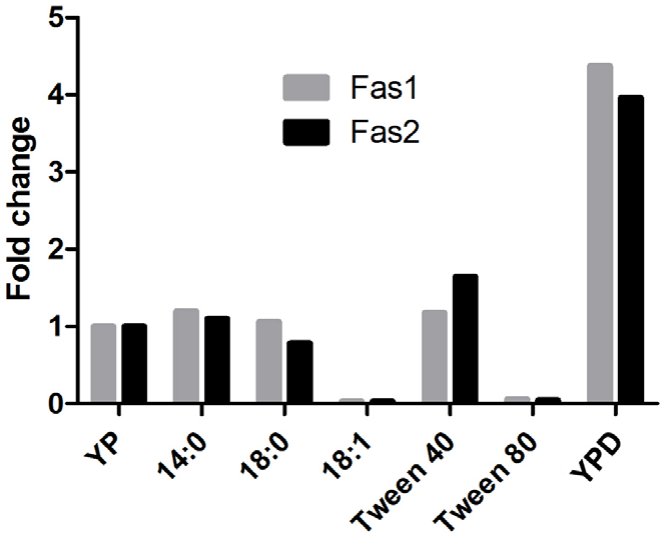
Relative expression levels of Fas1 and Fas2. Gene expression levels were determined by qRT-PCR in wild type *C. parapsilosis* in YP (1% yeast extract, 2% peptone), YPD with 1% (w/v) glucose (YPD), YP with 0.1% (w/v) myristic acid (14∶0), stearic acid (18∶0), or oleic acid (18∶1), and YP with 0.5% (v/v) Tween 40 or Tween 80. The cells were grown at 30°C for 20 hours. The results are the mean values from two experiments performed with triplicates.

To better understand the role of the fatty acid synthase genes (*CpFas1* and *CpFas2*) in response to carbon sources such as glucose and exogenous fatty acids, we used qRT-PCR to evaluate the expression of the genes in WT yeast. The genes were transcribed in medium without exogenous fatty acids (YP), indicating a basal requirement of *CpFas1* and *CpFas2* for cellular fatty acid synthesis (Figure 4). Expression of Fas1 and Fas2 was significantly elevated by the presence of glucose (YPD). Interestingly, we found that the unsaturated fatty acids oleic acid (18∶1) and Tween 80 down-regulated the expression of the fatty acid synthases while saturated fatty acid myristic (14∶0), stearic acid (18∶0), and Tween 40 did not. The expression of Fas1 and Fas2 genes in all conditions was balanced, suggesting that the FAS complex is coordinately regulated at transcriptional levels.

### Deletion of Fas2 Genes Affects Fatty Acid Compositions

We performed fatty acid profiling analysis of Fas2 KO and the WT grown in YPD, YPDT40 and YPDM (YPD plus 0.05% myristic acid). We found that the production of palmitic, palmitoleic, oleic, linoleic acids in the Fas2 KO was reduced (Table 2). Significantly, we found that the amount of saturated fatty acids (SFA) was elevated about 6.4 – 9.3% in the Fas2 KO. The ratio of SFA to UFA of the Fas2 KO was 12.74% compared with 5.03% from the WT when grown in YPDT40 and it was 12.27% compared with 6.17% from WT when grown in YPDM. The WT grown in YPD produced significantly higher amount of unsaturated fatty acids about 23.05% compared with 16.59% and 13.94% in the saturated fatty acid containing medium YPDT40 and YPDM, respectively (Table 2). This data suggests that Fas2 governs the fatty acid composition balance of *C. parapsilosis*.

**Table 2:** Fatty acid profiles (%) of the wild-type and Fas2 KO grown under different conditions.

Fatty acid	WT (YPDT40)	KO (YPDT40)	WT (YPDM)	KO (YPDM)	WT (YPD)
14∶0^a^	1.72	2.97	3.88	10.05	1.52
16∶0	49.83	44.09	39.33	37.83	39.36
16∶1	0.20	0.049	0.04	0.01	0.08
18∶0	31.79	45.58	42.80	44.53	36.02
18∶1	8.00	4.09	6.14	2.82	7.82
18∶2	8.39	3.14	7.75	4.70	15.16
20∶0	0.07	0.08	0.05	0.05	0.06
SFA^b^	83.41	92.72	86.06	92.47	76.95
UFA	16.59	7.28	13.94	7.53	23.05
SFA/UFA ratio	5.03	12.74	6.17	12.27	3.34

a 14∶0 = myristic acid, 16∶0 = palmitic acid, 16∶1 = palmitoleic acid, 18∶0 = stearic acid, 18∶1 = oleic acid, 18∶2 = linoleic acid, 20∶0 = eicosanoic acid.

b SFAs (saturated fatty acids) = 14∶0+16∶0+18∶0+20∶0; UFAs (unsaturated fatty acids) = 16∶1+18∶1+18∶2.

### Hypersensitivity of Fas2 KO to Human Serum

**Figure 5:**
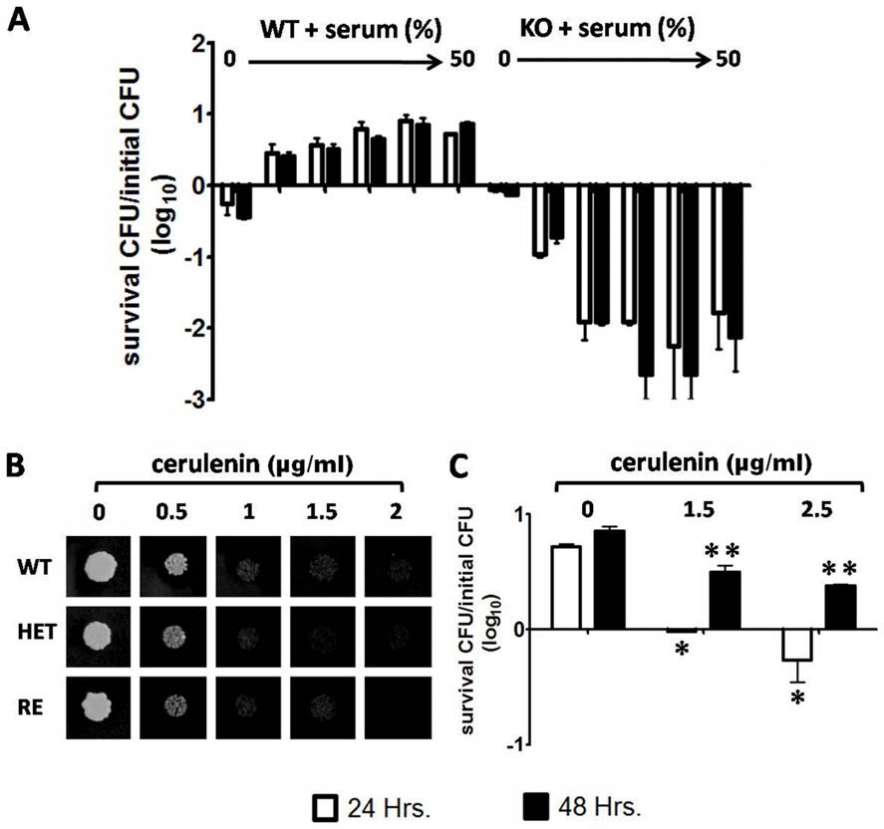
Growth of the wild type (WT) and knockout (KO) in PBS (0) or 10, 20, 30, 40, or 50% of human serum diluted in PBS (A). Susceptibility test of WT, HET, and RE strains with indicated concentrations of cerulenin (µg/ml) in YPD agar incubated at 30°C for 2 days (B). Inhibition of WT growth by indicated concentration of cerulenin in 50% human serum (C). The number of CFU was expressed as log10. Experiments were repeated twice with triplicates. Error bars indicate standard deviation. *P<0.01; **P<0.05 (ANOVA).

Human serum is a rich source of fatty acids. It contains approximately 80% of the saturated palmitic and stearic acids and the unsaturated linoleic and oleic acids [24]. We examined the growth of the WT and Fas2 mutant strains in different concentrations of heat-inactivated human serum. As shown in figure 5A, all the strains except the Fas2 KO were able to grow in different concentrations of human serum. The CFU of the WT grown in 10 to 50% human serum increased approximately 3 to 8 fold after 24 hours (Figure 5A). A longer incubation time (48 hours) did not significantly increase WT growth. Growth rates of HET and RE strains in 50% human serum were similar to WT growth (data not shown). In contrast, the Fas2 KO was hypersensitive to human serum as the number of cells decreased by >85% in 10 to 50% serum after 24 and 48 hours of incubation (Figure 5A).

Cerulenin is a natural antibiotic compound known to inhibit fatty acid synthase [17], [18]. We compared the activity of ceulenin on *C. parapsilosis* WT and the HET and RE mutants. The effect of cerulenin was similar on the different strains indicating that the deletion of a single Fas2 gene did not increase fungal susceptibility to the compound (Figure 5B). We determined that the MIC90 of cerulenin for the WT was 1.5 µg/ml. We next examined the impact of cerulenin on the growth of WT in 50% human serum. Interestingly, we found that exposure to cerulenin at or above the MIC (2.5 µg/ml) led to reduced growth of the WT at 24 hours and 48 hours of treatment (Figure 5C). We found that the antifungal activity of cerulenin is fungicidal as the number of CFU was decreasing after 24 hours of treatment (Figure 5C).

### *CpFas2* Is Required for Effective Biofilm Formation

**Figure 6:**
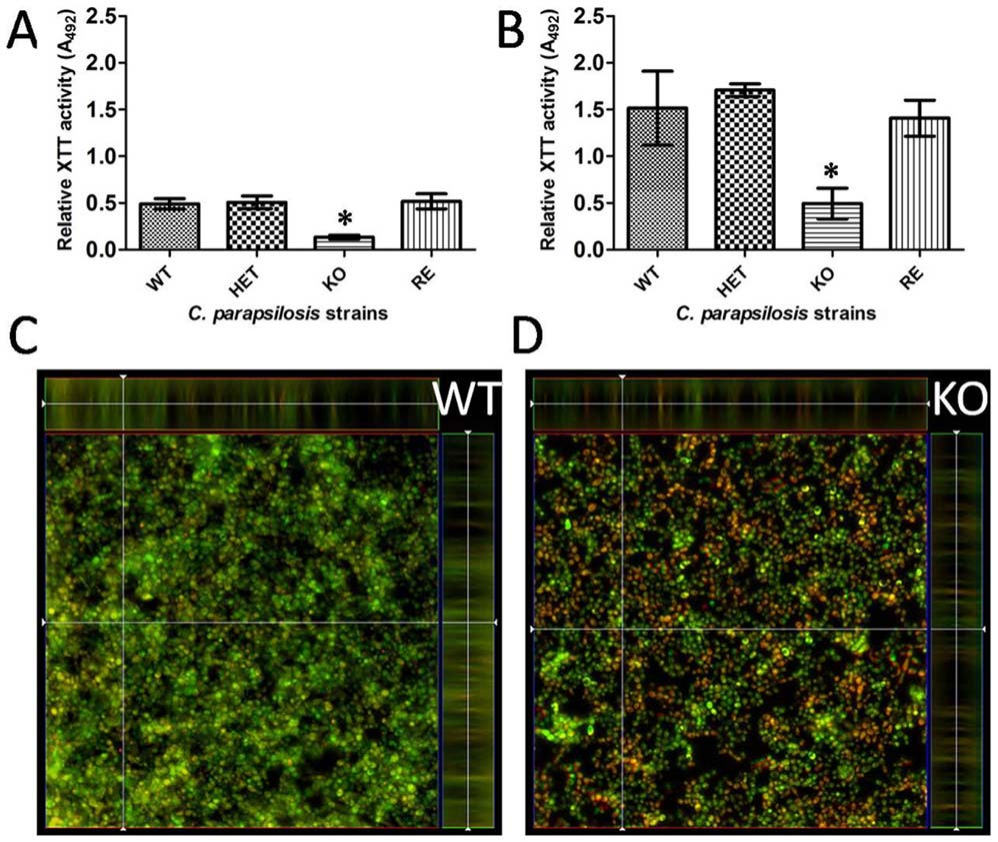
Comparison of biofilm formation of wild type (WT), heterozygous (HET), homozygous (KO), and reconstituted (RE) mutant strains on polysterene and silicone surfaces. Metabolic activity of the cells was measured by XTT assay on polysterene plates (A), and plates containing silicone disk (B). Microscopic analysis of biofilm structures of the WT (C) and KO (D) strains formed after 48 hours on polysterene plates. XTT assay was measured at 492 nm. Experiments were performed twice with triplicates that reproduced similar results. Error bars indicate standard deviation. *P<0.01 (ANOVA).

The involvement of *CpFas2* in biofilm formation on polysterene (96-well plate) and silicone sheet surfaces (24-well plate) was examined by a metabolic activity assay with XTT and by fluorescence microscopy. As shown in figure 6A and B the metabolic activity of the Fas2 KO strain was significantly decreased under biofilm conditions on both surfaces. The differences in biofilm structure between the WT and the Fas2 KO strains were further examined by fluorescence microscope in which the metabolically active cells were stained with FUN1 (red) and polysaccharides were stained with ConA Alexa fluor 488 conjugates (green). The biofilm formed by Fas2 KO yeast consisted of a single layer of metabolically active cells with minimal accumulation of polysaccharides (Figure 6D), whereas the WT strain formed a more complex structure with multiple layers of cells and polysaccharides (Figure 6C). The depth of the biofilm structures is demonstrated by the Z-stack analyses, which reveals the more complex nature of the WT biofilm. The HET and RE strains exhibited similar biofilm phenotypes to the WT strain (data not shown). Thus, the data suggests that the *CpFas2* is important for biofilm production.

### *CpFas2* Promotes Fungal Survival in Macrophages

**Figure 7:**
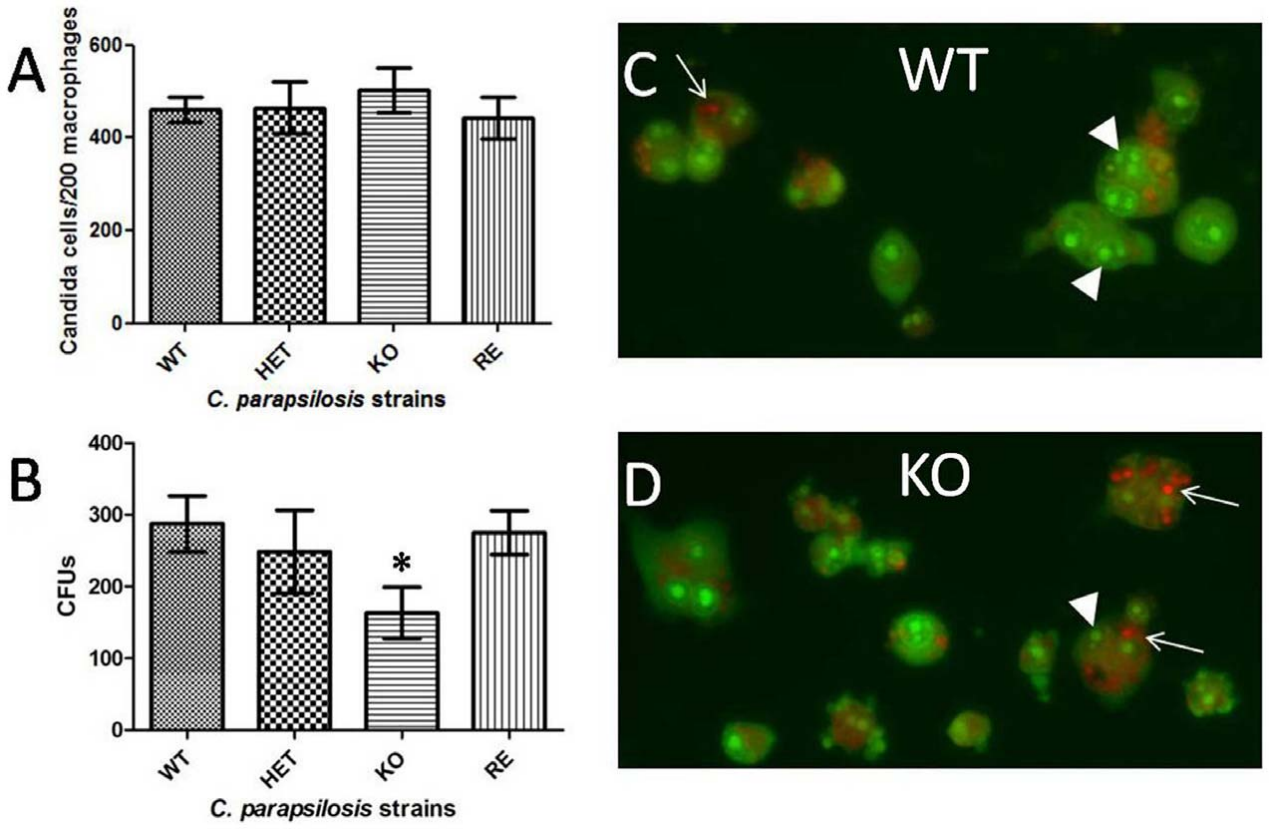
Phagocytosis and killing assays of the wild type (WT), heterozygous (HET), homozygous (KO), and reconstituted (RE) mutant strains with murine-like macrophages J774.16. Phagocytosis of *C. parapsilosis* strains by J774.16 (A). Determination of CFUs after 2 hour co-culture of yeast cells with J774.16 (B). Intracellular viability of yeast as determined by acridine and crystal violet staining of the WT (C) and the Fas2 KO strains (D) in the macrophages. The green yeast cells (arrow heads) are alive whereas the orange-red cells (arrows) are dead. Pictures are the merge of the red, green, and phase channels. Experiments were repeated at least twice and similar results were obtained. Error bars indicate standard deviation. *P<0.01 (ANOVA).

Phagocytosis of the Fas2 mutant strains was evaluated using the murine-like macrophage line J774.16. We found that deletion of the Fas2 genes did not alter the rate of phagocytosis of the *C. parapsilosis* strains (Figure 7A). However, the intracellular survival of Fas2 KO yeast was significantly reduced compared to WT or heterozygous cells. The intracellular survival of Fas2 KO yeast after 2 hours co-culture was ∼40% less than WT or strains with a single Fas2 gene (Figure 7B). This finding was supported by immunofluorescent analysis in which direct visualization of live (green) and dead (orange red) yeast cells inside the macrophages is achieved. Figure 7C and D demonstrates that significantly more WT cells survived than KO cells. Analysis of >200 macrophage in multiple fields in different quadrants on the slides revealed that ∼37.7% of WT cells was killed compared to ∼67.5% of KO. The intracellular survival of HET and RE yeast strains by this method was similar to WT yeast (data not shown).

### Deletion of Fas2 Enhances the Susceptibility of Yeast Cells to Stress Conditions

**Figure 8:**
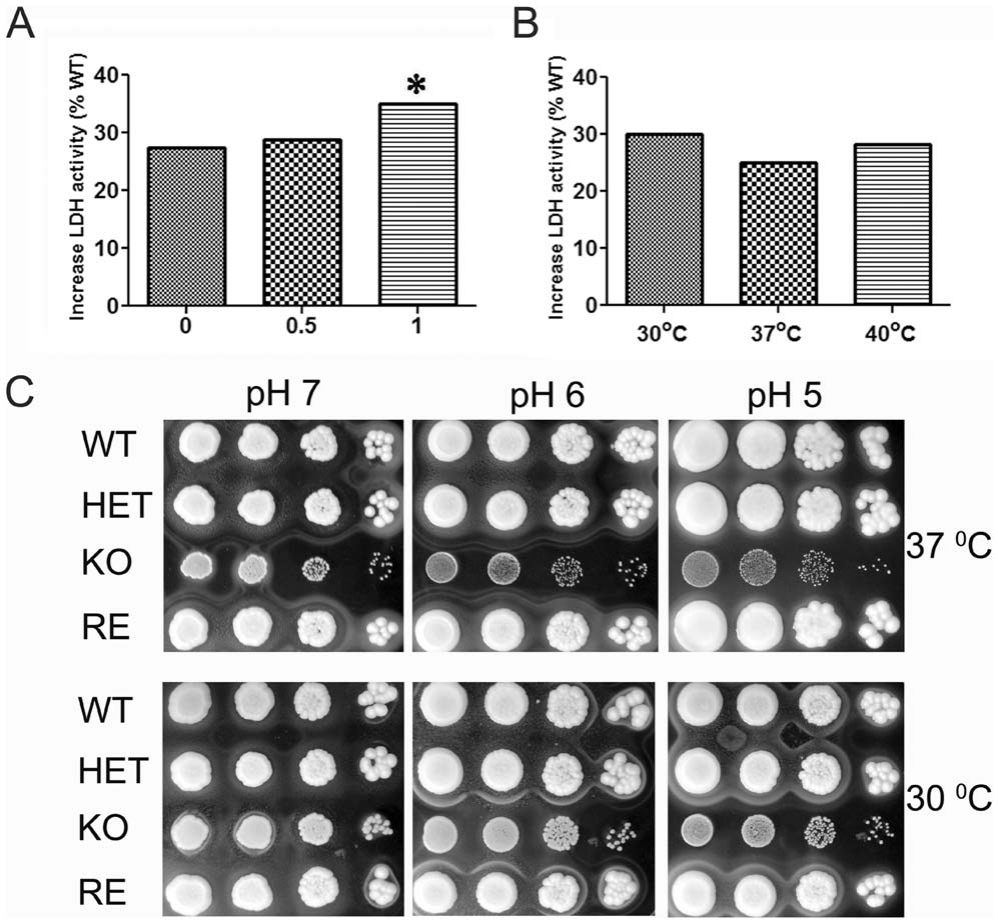
Lactate dehydrogenase (LDH) activity of Fas2 KO under stress conditions and growth of yeast cells in YPDT40 at different pH and temperature. LDH activity of Fas2 KO released after exposure of the yeast cells to 0, 0.5 and 1 mM of hydrogen peroxide at 37°C for 1 hour (A). LDH activity after incubation of yeast cells at 30°C, 37°C and 40°C for 1 hour (B). LDH activity of Fas2 KO was expressed as % increase compared to WT. The results are the average from two independent experiments performed in triplicate. Spot test of Fas2 mutant and wild type grown in YPDT40 at the indicated pH (C). *P<0.05 (ANOVA).

Since deletion of Fas2 KO led to significant clearance of yeast cells by J774.16 macrophages (Figure 7B), we tested the susceptibility of the yeast to different concentrations of hydrogen peroxide. Although the Fas2 KO growth was similar to WT strain in the assays with the addition of fatty acids, we found that the mutant had significantly higher (∼28%) LDH activity even in the absence of hydrogen peroxide compared to the wild type (Figure 8A). Addition of hydrogen peroxide further increased LDH activity, significantly at 1 mM hydrogen peroxide (Figure 8A). LDH activity of the Fas2 KO was also elevated when the yeast cells were incubated at 30°C and 40°C (Figure 8B). The Fas2 KO exhibited reduced growth in lower pH media (Figure 8C). The reduced growth was also observed at higher temperature (Figure 8C). These data indicated that Fas2 plays a role in stress response, presumably by governing the balance of fatty acids.

### *CpFas2* Is Required for Systemic Infection

**Figure 9:**
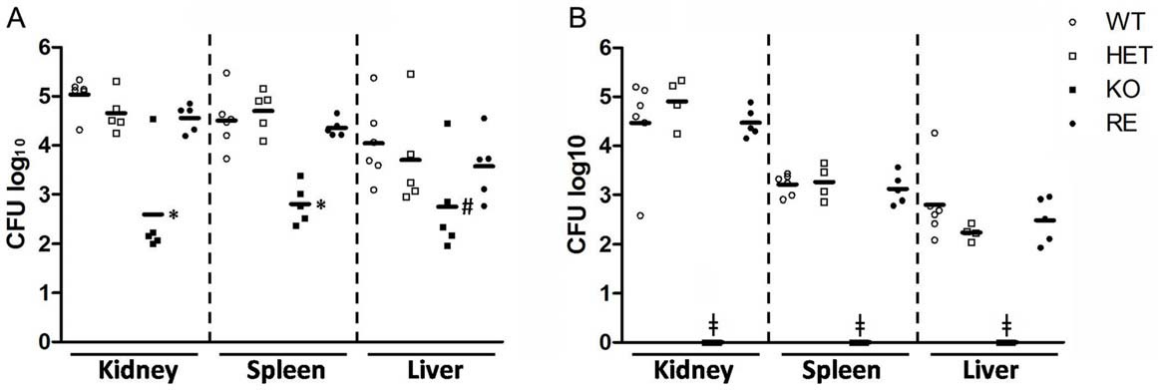
Intraperitoneal infection of A/J mice with wild type (WT), heterozygous (HET), homozygous (KO), or reconstituted (RE) yeasts. (A) CFUs in the kidney, spleen, and liver 3 days after intraperitoneal infection and (B) 5 days after infection. Each symbol represents 1 mouse. *P≤0.001, ^#^ *P*≤0.01 (Newman-Keuls). ^‡^, no detectable CFU of KO mutants.

AJ mice were inoculated intraperitoneally with either WT, RE, HET or KO cells and fungal burdens were examined 3 and 5 days after infection. The homozygous Fas2 KO cells were significantly less virulent, as demonstrated by the reduced kidney, spleen and liver CFUs compared to the CFUs of organs from mice infected with WT cells 3 days after infection (Figure 9A). In contrast to mice infected with WT or heterozygous yeast strains, mice challenged with the Fas2 KO strain had no yeast detectable in the organs examined at day 5 after infection (Figure 9B).

## Discussion

Fatty acids are major building blocks of cell membranes, which are products of cellular biosynthesis. *Candida* species such as *C. albicans*, *C. glabrata*, and *C. parapsilosis* produce a variety of hydrolytic enzymes that are involved in nutrient acquisition and virulence. We previously demonstrated that the secreted lipase of *C. parapsilosis* significantly impacts fungal growth and virulence [4], suggesting that host lipids are important nutritional resources. To further explore the role of fatty acids in the pathobiology of *C. parapsilosis*, we decided to study the affect of the Fas2 genes on growth and survival under various conditions.

Disruption of the Fas2 genes led to the generation of fatty acid-auxotrophic mutants (Fas2 KO strains), which were capable of growing in the presence of certain exogenous fatty acids. This result is consistent with data from a *C. albicans* Fas2 disruptant [14], [15]. We found that *C. parapsilosis* Fas2 KO growth was rescued by addition of certain saturated fatty acids such as myristic acid (14∶0) and palmitic acid (16∶0), but not stearic acid (18∶0) and the monounsaturated oleic acid (18∶1). The results suggest that saturated myristic and palmitic acids are the products of the FAS complex, and in *C. albicans* they appear to be the preferred substrates for subsequent de-saturation by Ole1 (delta (9) fatty acid desaturase) [25], [26]. In fact, supplementation of palmitoleic (16∶1) but not stearic and oleic acids rescues the growth of a *C. albicans* Ole1 repression mutant [26]. Interestingly, we also found that supplementation with 18-carbon fatty acids could not compensate for the lack of Fas2 activity. *C. parapsilosis* lipases have substrate specificity between C10-C16 [27]. To assess the role of lipase in the pathway of fatty acid acquisition for Fas2 processing, we tested the growth of Fas2 KO strain in the presence of intralipid and olive oil (data not shown), which are the substrates of secreted lipases. Since both of these mediums afforded normal growth of the Fas2 KO cells, the Fas2 disruptants were able to utilize the fatty acids liberated from lipase activity. These results indicate certain exogenous fatty acids can bypass the role of Fas2 genes for *C. parapsilosis* growth *in vitro* and demonstrate the essential roles of Fas2 genes for *de novo* fatty acid synthesis in medium lacking fatty acids.

Biofilm formation is an important virulence factor for fungal infection. The ability to form biofilm on indwelling medical devices is strongly correlated with fungal resistance to different antifungal treatments [28]. This is especially important for *C. parapsilosis*, since this pathogen is notorious for producing tenacious biofilms on catheters [2]. Interestingly, microarray studies have shown that *C. parapsilosis* Fas2 genes are upregulated during *in vitro* biofilm formation under hypoxic conditions [29]. Our results demonstrate that the Fas2 KO strain is significantly impaired in its capacity to form biofilms on polysterene and silicone surfaces, indicating the importance of *CpFas2* in biofilm production. In a similar manner, *Mycobacterium smegmatis* mutants lacking a chaperonin 60 (*GroEL1*) are deficient in mycolic acid production and are unable to effectively produce biofilms [30]. Interestingly, analysis of a Δ*groEL1* mutant proteome revealed a marked reduction in KasA and KasB components of the type II fatty-acid synthase complex involved in the synthesis of mycolic-acid precursors [30]. Since *CpFas2* is putatively involved in fatty acid elongation from shorter fatty acid precursors, we postulate that long carbon chain fatty acid products are required for biofilm matrix formation and also are involved in promoting membrane plasticity or fluidity. It could also be that a lack of essential fatty acids alters structural membrane complexity resulting in changes in the organization of membrane proteins. We evaluated the fatty acid compositions of the wild type and Fas2 KO grown under different growth conditions (Table 2). We found significant changes in ratio of saturated fatty acids and unsaturated fatty acids (Table 2). This suggests the Fas2 regulates the fatty acid compositions which correlate with the altered capacity of the disruptants to combat stress conditions and might also be important for cell adhesion during biofilm formation.

Eradication of *Candida* cells in the human host is largely dependent on the fungicidal activity of monocytes, such as neutrophils and macrophages [31]. Although deletion of *C. parapsilosis* Fas2 genes did not significantly enhance phagocytosis by the J774.16 macrophage-like cell line, their disruption enabled the macrophages to kill the fungus more efficiently. Changes in fatty acid compositions have been shown to affect *C. albicans* cell membrane fluidity [25]. Reduced membrane fluidity/stability could enhance the Fas2 KO susceptibility to reactive oxygen species secreted by the macrophages. We demonstrated that Fas2 KO is more susceptible to oxidative stress. The Fas2 KO exhibited a leaky phenotype and was defective in growth under stress conditions. Thus, we propose that organization of the cell membrane in the Fas2 KO was affected increasing its susceptible to oxygen species released from macrophages. Additionally, the lack of essential fatty acids generated by Fas2 could alter intracellular viability and proliferation as the Fas2 KO is auxotrophic for fatty acids. Moreover, deletion of Fas2 genes from *C. parapsilosis* significantly reduced the survival of the mutant cells during systemic infection. This is similar to what was shown for *C. albicans* Fas2 disruptants [14], [15].

To test the contribution of human serum fatty acids as an energy source for *C. parapsilosis* growth, the WT, HET, RE, and Fas2 KO strains were grown in different concentrations of human serum. Interestingly, the WT and heterozygous strains were able to utilize this medium for growth whereas the Fas2 disruptant failed to grow. Furthermore, the serum medium was toxic to the KO strain, reducing viability by > 85%. As a proof in principle for assessing the importance of Fas2 as a drug target, we determined the impact of Fas2 inhibition in *C. parapsilosis* by the Fas2 inhibitor cerulenin. Cerulenin significantly impeded the growth of WT and heterozygous strains. Moreover, cerulenin reduced *C. parapsilosis* WT growth in human serum, demonstrating that Fas2 inhibition is effective in this fatty acid rich setting. This result is in contrast with work in bacteria where human serum can overcome the effects of bacterial fatty acid synthase inhibition [12]. Hence, our findings further support the targeting of fungal Fas2 genes for antifungal drug development.

Fungal FAS genes (Fas1 and Fas2) are considered to be housekeeping genes. They are constitutively expressed and are subjected to elaborate regulation by several factors to coordinately control the expression of both subunits [5]. Our results demonstrate that the transcriptional expression of Fas1 and Fas2 genes was coordinately expressed in response to medium cues. Expression of the fatty acid synthase genes was increased in response to glucose and decreased in response to fatty acid availability, indicating their roles of fatty acid *de novo* synthesis. The up-regulation of fatty acid synthesis genes of *C. parapsilosis* such as Acc1 (acyl-CoA carboxylase), Fas1, Fas2, and Ole1 in response to biofilm formation and hypoxia has been shown microarray study [29]. In the current study, we demonstrate that a fatty acid synthesis pathway of the human pathogen *Candida parapsilosis* plays a crucial role in fungal growth during infection and we provide evidence that *CpFas2* may be an important candidate for fungal infection treatment.
